# Supplementary material for: Polymorphisms in Pfkelch13 domains before and after the introduction of artemisinin-based combination therapy in Southwest Nigeria
Source: PLoS One. 2025 Mar 31;20(3):e0316479. doi: 10.1371/journal.pone.0316479 (PMC11957316; doi:10.1371/journal.pone.0316479)

Samples: 12014
Bases: 1074
Average spacing: 12

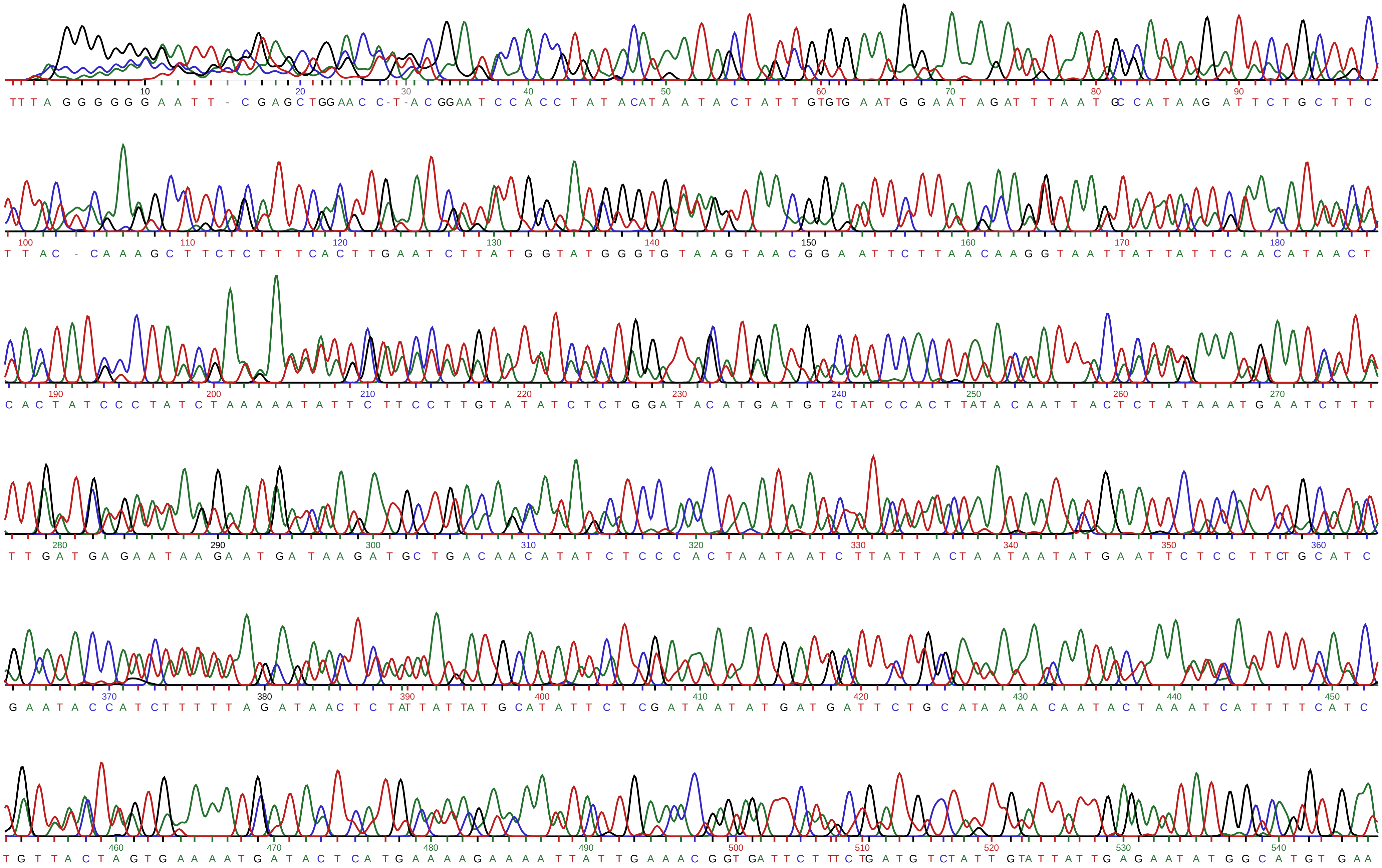

Samples: 12014
Bases: 1074
Average spacing: 12

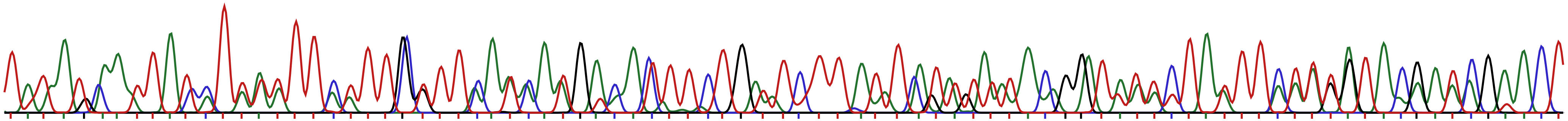

550 560 570 580 590 600 610 620 630

T A T A G A A T T A T C T T A T T T C T T T G T T T C A T C A T G A C A C T T C T G T T C T T A T T A T A T T T A C G G T A T T C T A T T T C T T T T A T A C G A T C G A A C T

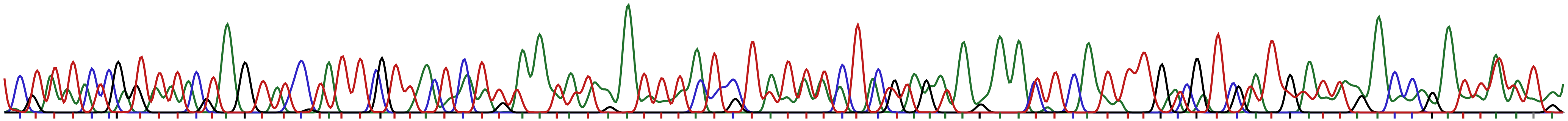

640 650 660 670 680 690 700 710 720

C T T T C CG T T T C T A G T T C TAT T G T T A T C T T A A T A T A A T T T A T C T A T T T C T C T A A A A G A A T T C A T T T G C G T C A T G A T T A A C C G A T T A A - A

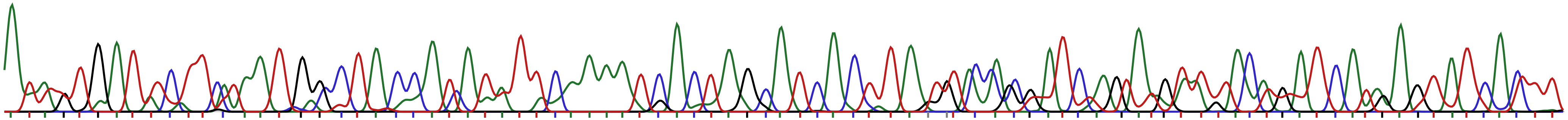

730 740 750 760 770 780 790 800 810

A T A G T G A T T C T T C A A T G G C T A C C A T A T A T T C A A A A T C A C T A G C A T C A C T T A - -T C A C G A T C A G A T G T T T A C T G A T C A T G A G T A T C A C T T

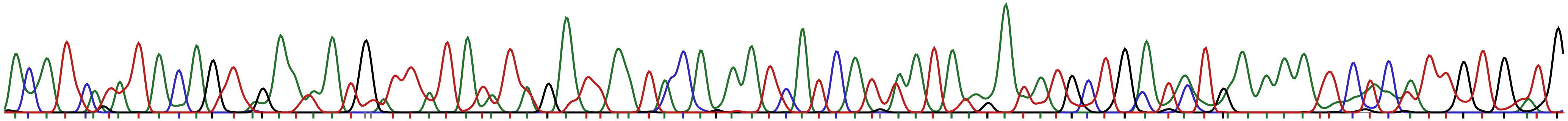

820 830 840 850 860 870 880 890 900

A C A T CA T A T A C A G T AG A T A A T - - T T A T A T A T A G A T T A A T A C A G A A T C A T C A T- A A T A A G A T A T G C T G A T A T G A A A A T T C T C A T T G T G A T G

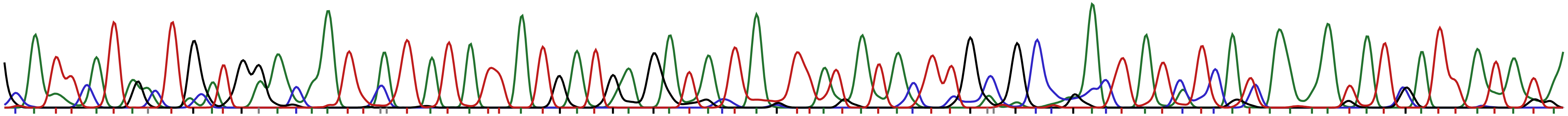

910 920 930 940 950 960 970 980

C A T T A T A - T G A T G - A C A A T T - - T A T A T T A T G A T G A G A T A C T A G T T A T A T A C T T G - - G C C G A C T A T C T C A T A A A A T A T G A T T A T A T A

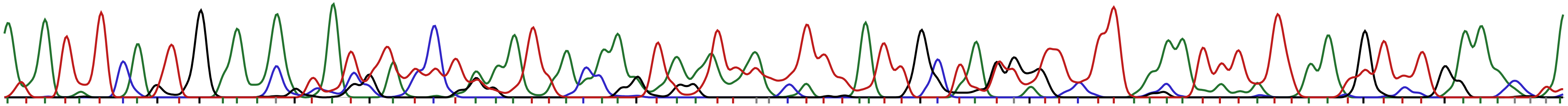

990 1000 1010 1020 1030 1040 1050 1060 1070

A T A T A T C A G T G A A A - G T A T G A T C T A A A T T A C A A G T A A T T - - C T T T A A T T G C T A T - G T T C T T A A A A T T T A T T A A T G T T T G A A A - - - A

Samples:	12014
Bases:	1074
Average spacing:	12

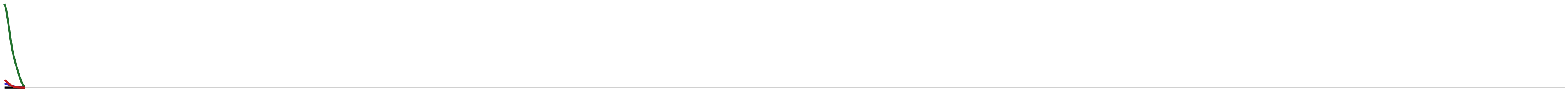

Supplement: Supporting information 2 — (ZIP) [file pone.0316479.s002.zip › 052KN2F_PREMIX_Plate_KELCH2_A07.pdf]
